# A spatially integrated framework for assessing socioecological drivers of carnivore decline

**DOI:** 10.1111/1365-2664.13072

**Published:** 2018-01-15

**Authors:** Nicolás Gálvez, Gurutzeta Guillera‐Arroita, Freya A. V. St. John, Elke Schüttler, David W. Macdonald, Zoe G. Davies

**Affiliations:** ^1^ Durrell Institute of Conservation and Ecology (DICE) School of Anthropology and Conservation University of Kent Canterbury Kent UK; ^2^ Department of Natural Sciences Centre for Local Development Pontificia Universidad Católica de Chile Villarrica Chile; ^3^ Fauna Australis Wildlife Laboratory School of Agriculture and Forestry Sciences Pontificia Universidad Católica de Chile Santiago de Chile Chile; ^4^ School of BioSciences University of Melbourne Parkville Vic. Australia; ^5^ School of Environment, Natural Resources & Geography Bangor University Bangor UK; ^6^ Department of Conservation Biology UFZ – Helmholtz Centre for Environmental Research GmbH Leipzig Germany; ^7^ Wildlife Conservation Research Unit Department of Zoology University of Oxford The Recanati‐Kaplan Centre Tubney OX UK

**Keywords:** camera trapping, conservation, güiña, habitat fragmentation, habitat loss, human–wildlife coexistence, illegal killing, kodkod, multiseason occupancy modelling, randomised response technique

## Abstract

1. Habitat loss, fragmentation and degradation are key threats to the long‐term persistence of carnivores, which are also susceptible to direct persecution by people. Integrating natural and social science methods to examine how habitat configuration/quality and human–predator relations may interact in space and time to effect carnivore populations within human‐dominated landscapes will help prioritise conservation investment and action effectively.
2. We propose a socioecological modelling framework to evaluate drivers of carnivore decline in landscapes where predators and people coexist. By collecting social and ecological data at the same spatial scale, candidate models can be used to quantify and tease apart the relative importance of different threats.
3. We apply our methodological framework to an empirical case study, the threatened güiña (*Leopardus guigna*) in the temperate forest ecoregion of southern Chile, to illustrate its use. Existing literature suggests that the species is declining due to habitat loss, fragmentation and persecution in response to livestock predation. Data used in modelling were derived from four seasons of camera‐trap surveys, remote‐sensed images and household questionnaires.
4. Occupancy dynamics were explained by habitat configuration/quality covariates rather than by human–predator relations. Güiñas can tolerate a high degree of habitat loss (>80% within a home range). They are primarily impacted by fragmentation and land subdivision (larger farms being divided into smaller ones). Ten per cent of surveyed farmers (*N* = 233) reported illegally killing the species over the past decade.
5. *Synthesis and applications*. By integrating ecological and social data, collected at the same spatial scale, within a single modelling framework, our study demonstrates the value of an interdisciplinary approach to assessing the potential threats to a carnivore. It has allowed us to tease apart effectively the relative importance of different potential extinction pressures for the güiña (*Leopardus guigna*), make informed conservation recommendations and prioritise where future interventions should be targeted. We have identified that human‐dominated landscapes with large intensive farms can be of conservation value, as long as an appropriate network of habitat patches is maintained within the matrix. Conservation efforts to secure the long‐term persistence of the species should focus on reducing habitat fragmentation rather than human persecution.

## Abstract 2

By integrating ecological and social data, collected at the same spatial scale, within a single modelling framework, our study demonstrates the value of an interdisciplinary approach to assessing the potential threats to a carnivore. It has allowed us to tease apart effectively the relative importance of different potential extinction pressures for the güiña (*Leopardus guigna*), make informed conservation recommendations and prioritise where future interventions should be targeted. We have identified that human‐dominated landscapes with large intensive farms can be of conservation value, as long as an appropriate network of habitat patches is maintained within the matrix. Conservation efforts to secure the long‐term persistence of the species should focus on reducing habitat fragmentation rather than human persecution.

## 1 INTRODUCTION

Land‐use change is one of the greatest threats facing terrestrial biodiversity globally (Sala et al., 2000), as species persistence is negatively influenced by habitat loss, fragmentation, degradation and isolation (Henle, Lindenmayer, Margules, Saunders, & Wissel, 2004). In general, species characterised by a low reproductive rate, low population density, large individual area requirements or a narrow niche are more sensitive to habitat loss and fragmentation (Fahrig, 2002; Henle, Davies, Kleyer, Margules, & Settele, 2004) and, therefore, have a higher risk of extinction (Purvis, Gittleman, Cowlishaw, & Mace, 2000). Consequently, many territorial carnivores are particularly vulnerable to land‐use change. Furthermore, the disappearance of such apex predators from ecosystems can have substantial cascading impacts on other species (Estes et al., 2011; Ripple et al., 2014).

Additionally, in human‐dominated landscapes, mammal populations are threatened directly by the behaviour of people (Ceballos, Ehrlich, Soberon, Salazar, & Fay, 2005). For instance, larger species (body mass >1 kg) are often persecuted because they are considered a pest, food source or marketable commodity (Woodroffe, Thirgood, & Rabinowitz, 2005). Carnivores are especially vulnerable to persecution after livestock predation, attacks on humans or as a result of deep‐rooted social norms or cultural practices (Inskip & Zimmermann, 2009; Marchini & Macdonald, 2012; Treves & Karanth, 2003). Indirectly, many mammals are also threatened by factors such as the introduction of invasive plant species, which reduce habitat complexity (Rojas et al., 2011), and domestic pets, which can transmit diseases or compete for resources (Hughes & Macdonald, 2013).

To ensure the long‐term future of carnivore populations within human‐dominated landscapes outside protected areas, it is imperative that we identify potential ecological and social drivers of species decline and assess their relative importance (Redpath et al., 2013). For example, it is essential to disentangle the impacts of habitat loss and fragmentation on a species, as the interventions required to alleviate the pressures associated with the two processes are likely to be different (Fahrig, 2003; Fischer & Lindenmayer, 2007). If habitat loss is the dominant issue causing population reduction, then large patches may need to be protected to ensure long‐term survival, whereas a certain configuration of remnant vegetation may be required if fragmentation is the main threat. At the same time, it is important to understand if, how and why people persecute species, if conservationists are to facilitate human‐wildlife coexistence (St John, Keane, & Milner‐Gulland, 2013). However, there is a paucity of interdisciplinary research that evaluates explicitly both ecological and social drivers of species decline in a single coherent framework, across geographic scales pertinent to informing conservation decision‐making (Dickman, 2010).

From an ecological perspective, data derived from camera traps and analysed via occupancy models are widely used to study carnivores over large geographic areas (Burton et al., 2015; Steenweg et al., 2016). Occupancy modelling offers a flexible framework that can account for imperfect detection and missing observations, making it highly applicable to elusive mammals of conservation concern (MacKenzie, Nichols, Hines, Knutson, & Franklin, 2003; MacKenzie & Reardon, 2013). Monitoring population dynamics temporally, and identifying the factors linked to any decline, is critical for management (Di Fonzo, Collen, Chauvenet, & Mace, 2016). For this reason, dynamic (i.e. multiseason) occupancy models are particularly useful because they examine trends through time and can be used to ascertain the drivers underlying observed changes in occupancy (MacKenzie et al., 2003, 2006). Similarly, there are a range of specialised social science methods for asking sensitive questions that can be used to yield valuable information on human behaviour, including the illegal killing of species (Nuno & St. John, 2015). One such example is the unmatched count technique, which has recently been used to examine the spatial distribution of hunting and its proximity to Serengeti National Park, Tanzania (Nuno, Bunnefeld, Naiman, & Milner‐Gulland, 2013) and bird hunting in Portugal (Fairbrass, Nuno, Bunnefeld, & Milner‐Gulland, 2016). Another method is the randomised response technique (RRT), previously used to estimate the prevalence of predator persecution in South Africa (St John et al., 2012) and vulture poisoning in Namibia (Santangeli, Arkumarev, Rust, & Girardello, 2016).

In this paper, we propose an integrated socioecological modelling framework that draws together these natural and social science methods to examine how habitat configuration/quality and “human–predator relations” (Pooley et al., 2016) may interact in space and time to effect carnivore populations across a human‐dominated landscape. An important aspect of the approach is that the social and ecological data are collected at a matched spatial scale, allowing different potential drivers of decline to be contrasted and evaluated. We showcase the approach using the güiña (*Leopardus guigna*), a felid listed as Vulnerable on the International Union for Conservation of Nature (IUCN) Red List, as a case study species. Specifically, we use data derived from multiseason camera‐trap surveys, remote‐sensed images and a household questionnaire which uses RRT to estimate prevalence and predictors of illegal killing. The outputs from our framework provide a robust evidence base to direct future conservation investment and efforts.

## 2 MATERIALS AND METHODS

### 2.1 Integrated socioecological framework

**Figure 1:**
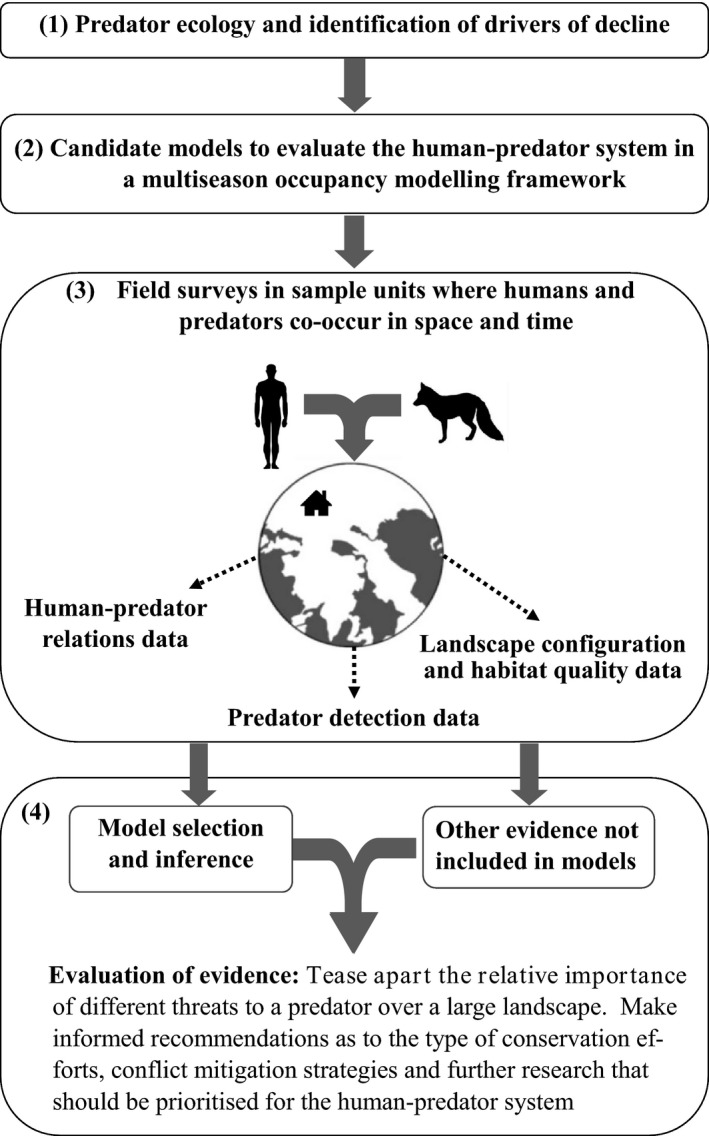
Integrated socioecological modelling framework to assess drivers of carnivore decline in a human‐dominated landscape

Our proposed framework comprises four stages (Figure 1). The first step is to gather information on the ecology of the species and likely drivers of decline, including habitat configuration/quality issues (e.g. habitat loss, habitat fragmentation and presence/absence of habitat requirements) and human–predator relations (e.g. species encounter frequency and livestock predation experiences), that require evaluation. The best available information can be acquired from sources such as peer reviewed and grey literature, experts and IUCN Red List assessments. The next task, step two, is to define a suite of candidate models a priori to assess and quantify the potential social and ecological predictors on species occupancy dynamics. Dynamic occupancy models estimate parameters of change across a landscape, including the probability of a sample unit (SU) becoming occupied (local colonisation) or unoccupied (local extinction) over time (MacKenzie et al., 2006).

The third step involves the collection of ecological and social data in SUs distributed across the landscape, to parametise the models. Camera‐trap survey effort allocation (i.e. the number of SUs that need to be surveyed) for occupancy estimation can be determined a priori using freely available tools (Gálvez, Guillera‐Arroita, Morgan, & Davies, 2016). The final stage is the evaluation of evidence, using standard model selection methods (Burnham & Anderson, 2002) to establish which of the social and ecological variables within the candidate models are indeed important predictors of occupancy and to contrast their relative importance. Results from the models can be contextualised with additional supporting evidence not embedded in the models to inform where conservation action should be directed. For instance, during questionnaire delivery, valuable qualitative data may be recorded that provides in‐depth insights related to the human–predator system (e.g. Inskip, Fahad, Tully, Roberts, & MacMillan, 2014).

### 2.2 Study species and system

The güiña is the smallest neotropical felid (<2 kg) (Napolitano, Gálvez, Bennett, Acosta‐Jamett, & Sanderson, 2015). It is thought to require forest habitat with dense understorey and the presence of bamboo (*Chusquea* spp.) (Dunstone et al., 2002; Nowell & Jackson, 1996) but is also known to occupy remnant forest patches within agricultural areas (Acosta‐Jamett & Simonetti, 2004; Fleschutz et al., 2016; Gálvez et al., 2013; Sanderson, Sunquist, & Iriarte, 2002; Schüttler et al., 2017). Güiñas are considered pests by some people as they can predate chickens and, while the extent of persecution has not been formally assessed, killings have been reported (Gálvez et al., 2013; Sanderson et al., 2002). Killing predominately occurs when the felid enters a chicken coop (Gálvez & Bonacic, 2008). Due to these attributes, the species makes an ideal case study to explore how habitat configuration/quality and human–predator relations may interact in space and time to influence the population dynamics of a threatened carnivore existing in a human‐dominated landscape.

**Figure 2:**
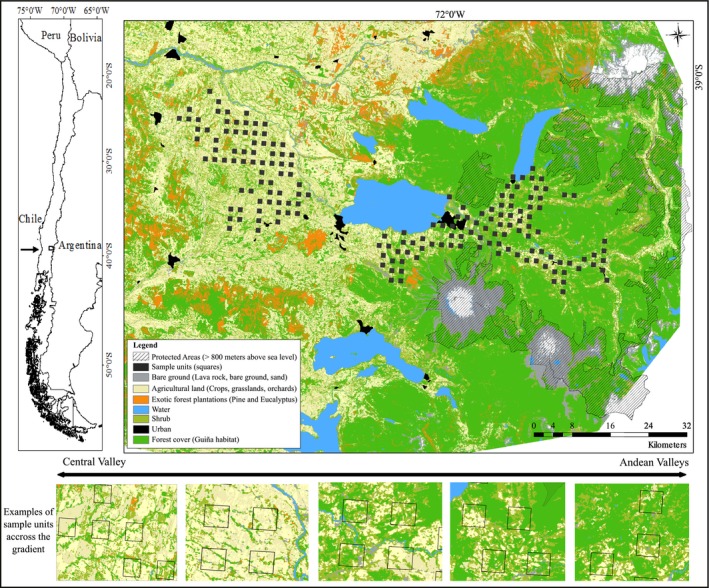
Distribution of landcover classes and protected areas across the study landscape in southern Chile, including the forest habitat of our case study species, the güiña (*Leopardus guigna*). The two zones within which the 145 sample units (SU: 4 km^2^) were located are indicated, 73 SUs in the central valley (left squares) and 72 within the Andes (right squares). Illustrative examples of the variation in habitat configuration within SUs across the human‐domination gradient are provided (bottom of image)

The study was conducted in the Araucanía region in southern Chile (Figure 2), at the northern limit of the South American temperate forest ecoregion (39°15′S, 71°48′W; Armesto, Rozzi, Smith‐Ramírez, & Arroyo, 1998). The system comprises two distinct geographical sections common throughout Southern Chile: the Andes mountain range and central valley. Land use in the latter is primarily intensive agriculture (e.g. cereals, livestock and fruit trees) and urban settlements, whereas farmland in the Andes (occurring <600 m.a.s.l) is less intensively used and surrounded by tracks of continuous forest on steep slopes and protected areas (>800 m.a.s.l). The natural vegetation across the study landscape consists of deciduous and evergreen *Nothofagus* forest (Luebert & Pliscoff, 2006), which remains as a patchy mosaic in agricultural valleys and as continuous tracts at higher elevations within the mountains (Miranda, Altamirano, Cayuela, Pincheira, & Lara, 2015).

### 2.3 Data collection

#### 2.3.1 Predator detection/non‐detection data

We obtained predator detection/non‐detection data via a camera‐trap survey. Potential SUs were defined by laying a grid of 4 km^2^ across the study region, representing a gradient of forest habitat fragmentation due to agricultural use and human settlement below 600 m.a.s.l. The size of the SUs was informed by mean observed güiña home range size estimates of collared individuals in the study area (MCP 95% mean = 270 ± 137 ha; Schüttler et al., 2017).

In this study system, detectability was modelled based on the assumption that a 2‐day survey block is a separate independent sampling occasion. This time threshold was chosen because initial observations of collared individuals indicated that they did not stay longer than this time in any single location (E. Schüttler et al. unpublished data). Minimum survey effort requirements (i.e. number of SUs and sampling occasions) were determined following Guillera‐Arroita, Ridout, and Morgan (2010), using species‐specific parameter values from Gálvez et al. (2013) and a target statistical precision in occupancy estimation of *SE* < 0.075. A total of 145 SUs were selected at random from the grid of 230 cells, with 73 and 72 SUs located in the central valley and Andes mountain valley respectively (Figure 2). The Andean valleys were surveyed for four seasons (summer 2012, summer 2013, spring 2013 and summer 2014), while the central valley was surveyed for the latter three seasons. A total of four rotations (i.e. blocks of camera traps) were used to survey all SUs within a 100‐day period each season. Detection/non‐detection data were thus collected for 20–24 days per SU, resulting in 10–12 sampling occasions per SU. Two camera traps (Bushnell ™trophy cam 2012) were used per SU, positioned 100–700 m apart, with a minimum distance >2 km between camera traps in adjacent SUs. The detection histories of both camera traps in a SU were pooled, and camera‐trap malfunctions or thefts (five in total) were treated as missing observations.

#### 2.3.2 Habitat configuration/quality data

The extent of habitat loss and fragmentation were evaluated using ecologically meaningful metrics which have been reported in the literature as being relevant to güiñas, using either field or remote‐sensed landcover data (Table 1, Appendix S1 and Table S1). The metrics were measured within a 300 ha circular buffer, centred on the mid‐point between both cameras in each SU using FRAGSTATS 4.1 (McGarigal, Cushman, Neel, & Ene, 2002). Habitat quality surrounding a camera trap might influence species activity (Acosta‐Jamett & Simonetti, 2004). We collected data on a number of variables within a 25‐m radius around each camera trap (Table S1), as this is deemed to be the area over which localised conditions may influence species detectability. The habitat quality data from both camera traps in each SU were pooled, and the median was used if values differed.

**Table 1:** Habitat configuration/quality and human relation predictors evaluated when modelling initial occupancy (ψ_1_), colonisation (γ), extinction (ε) and detection (*p*) probability parameters of multiseason camera‐trap güiña (*Leopardus guigna*) surveys. Further details can be found in Appendix S1, S2 and Table S1

Parameter	Predictor	Abbreviation in models
	*Habitat configuration*	
ψ_1_, ε, γ	Percentage of forest cover/habitat^a^	Forest
ψ_1_, ε, γ	Percentage of shrub cover/marginal habitat	Shrub
ψ_1_, ε, γ	Number of forest patches	PatchNo
ψ_1_, ε, γ	Shape index forest patches	PatchShape
ψ_1_, ε, γ	Forest patch size area^b^	PatchAreaW
ψ_1_, ε, γ	Forest patch continuity^b^	Gyration
ψ_1_, ε, γ	Edge length of forest land cover class	Edge
ψ_1_, ε, γ	Landscape shape index of forest^c^	LSI
ψ_1_, ε, γ	Patch cohesion^b^	COH
	*Human–predator relation*	
ψ_1_, ε	Land subdivision	Subdivision
ψ_1_, ε	Intent to kill (hypothetical scenario questions)	Intent
ψ_1_, ε	Predation	Predation
ψ_1_, ε	Frequency of predation	FQPredation
ψ_1_, ε, *p*	Frequency of encounter^d^	FQEncounter
ψ_1_, ε	Number of dogs	Dogs
	*Habitat quality*	
*p*	Bamboo density (*Chusquea* spp.)	Bamboo
*p*	Density of understorey	Understorey
*p*	Sample Unit rotation block	Rotation
*p*	Intensity of livestock activity	Livestock
*p*	Intensity of logging activity	Logging
*p*	Water availability	Water

a Pools together all forest types: old growth, secondary growth and wetland forest.

b Predictor excluded due to collinearity with percentage of forest cover (Pearson's │*r*│ > .7).

c Predictor excluded due to collinearity with number of forest patches (Pearson's │*r*│>.7).

d Predictor also fitted with detection probability.

#### 2.3.3 Human–predator relations data

Between May and September 2013, the questionnaire (Appendix S2) was administered face‐to‐face by NG who is Chilean and had no previous interaction with respondents. All SUs contained residential properties and one or two households closest to the camera‐trap locations were surveyed (mean number of households per km^2^ across the study landscape: 3.4; range: 1.4–5.1 from INE, 2002). For each household, the family member deemed to be most knowledgeable with respect to farm management and decision‐making was surveyed. The questionnaire gathered data on sociodemographic/economic background, güiña encounters, livestock ownership, frequency of livestock predation by güiñas and ownership of dogs on the land parcel. To measure tolerance to livestock predation, participants were asked how they would respond to different scenarios of livestock loss (mortality of 2, 10, 25, 50, >50 animals), with one possible option explicitly stating that they would kill güiña. These data were also used as predictors of killing behaviour in the RRT analysis (see below). The questionnaire was piloted with 10 local householders living outside the SUs; their feedback was used to improve the wording, order and time‐scale of predation and encounter questions.

The potential occupancy model predictors (Tables 1 and S1, Appendix S2) were calculated per SU. Where questionnaire responses differed within a SU (e.g. one household report predation and the other did not), the presence of the event (e.g. predation) was used as a covariate for that particular SU. For all quantitative measures, and when both respondents report the event (e.g. frequency of predation), median values were used.

#### 2.3.4 Illegal killing prevalence across the landscape (other evidence)

As it is illegal to kill güiñas in Chile (Law 19.473 Ministry of Agriculture), RRT (Nuno & St. John, 2015) was used to ask this sensitive question as part of the questionnaire (Appendix S2). Since RRT, like other methods for asking sensitive questions, requires a large sample size for precise estimation of behaviour prevalence (Nuno & St. John, 2015), we pooled RRT data from all participants to estimate the prevalence of illegal güiña killing across the landscape over the past decade. We explored predictors that might explain this human behaviour (St John et al., 2012).

Randomised response technique data were bootstrapped 1,000 times to obtain a 95% confidence interval. We tested seven non‐correlated predictors of illegal güiña killing: age, income, frequency of güiña encounters, number of chickens owned (all continuous variables standardised to *z* scores), economic dependency on their land parcel (1 = no dependency; 2 = partial dependency; 3 = complete dependency), knowledge of the güiña's legal protection status (0 = hunting prohibited; 1 = do not know; 2 = hunting permitted) and intention to kill a güiña under a hypothetical predation scenario (0 = do nothing; 1 = manage güiña; 2 = kill güiña; Appendix S2). We used r (version 3.2.3; R Core Team, 2014) to run the RRlog function of the package RRreg (version 0.5.0; Heck & Moshagen, 2016) to conduct a multivariate logistic regression using the model for “forced response” RRT data. We fitted a logistic regression model with the potential predictors of killing behaviour and evaluated their significance with likelihood ratio tests (LRT ∆*G* ^2^). Odds ratios and their confidence values are presented for model covariates.

### 2.4 Integrated socioecological modelling

First, we evaluated the existence of spatial autocorrelation with detection/non‐detection data for each SU, using Moran's *I* index based on similarity between points (Dormann et al., 2007). We used a fixed band distance of 3 km from the mid‐point of camera traps, equating to an area three times larger than a güiña home range.

We fitted models of occupancy dynamics (MacKenzie et al., 2003) using PRESENCE, which obtains maximum likelihood estimates via numerical optimisation (Hines, 2006). The probabilities of initial occupancy (ψ), colonisation (γ), local extinction (ε) and detection sites (*p*) were used as model parameters. We conducted a preliminary investigation to assess whether a base model structure with Markovian dependence was more appropriate for describing seasonal dynamics, rather than assuming no occupancy changes occur or that changes happen at random (MacKenzie et al., 2006). Once the best model structure had been determined, we then fitted models with habitat configuration/quality and human–predator predictors.

A total of 15 potential model predictors were tested for collinearity and, in instances where variables were correlated (Pearson's/Spearman's│*r*│>.7), we retained the covariate that conferred greater ecological/social meaning and ease of interpretation (Table 1 and Table S1). All continuous variables, except percentages, were standardised to *z*‐scores. We approached model selection by increasing model complexity gradually, fitting predictors for each model parameter separately and assessing model performance using Akaike's information criterion (AIC). Models that were within <2 ∆AIC were considered to have substantial support (Burnham & Anderson, 2002), and thus, these predictors were selected and used in the next step in a forward manner (e.g. Kéry, Guillera‐Arroita, & Lahoz‐Monfort, 2013). To prevent over fitting (Burnham & Anderson, 2002), we kept models with only one predictor per parameter, with the exception of one model which evaluated the additive effect of shrub and forest cover (shrub is a marginal habitat for the study species; Dunstone et al., 2002).

A set of detection models was fitted using the best base structure. Subsequently, we evaluated models that included habitat configuration/quality and human–predator relations data to test its effect on initial occupancy (ψ_1_), while keeping colonisation and extinction specific. The best initial occupancy and detection models were then used to add further complexity to the colonisation and extinction components. We fitted all predictors for extinction. However, we assume that colonisation between seasons is primarily influenced by habitat configuration/quality variables, rather than human–predator relations. To explore the candidate model space, we worked on the structure for extinction probability followed by colonisation and then repeated the process vice versa (Kéry et al., 2013). A constant or null model was included in all candidate model sets. Models with convergence problems or implausible parameter estimates (i.e. very large estimates and *SE*s) were eliminated from each set.

Goodness‐of‐fit was evaluated by bootstrapping 5,000 iterations (MacKenzie & Bailey, 2004) in the r package AICcmodavg. This test provides a model fit statistic based on consideration of the data from all seasons at once (*p*‐Global) as well as separate statistics for each season. We used the predict function in r package unmarked (Fiske & Chandler, 2011) to produce plots of estimated relationships with the predictors and derive estimates of occupancy for each of the seasons.

All aspects of this project were approved by the School of Anthropology and Conservation Research and Research Ethics Committee, University of Kent as well as the Villarrica Campus Committee of the Pontificia Universidad Católica de Chile.

## 3 RESULTS

### 3.1 Habitat configuration/quality data

Across the landscape, variation in the degree of habitat loss and fragmentation was substantial. Forest cover in SUs ranged from 1.8% to 76% (*M* = 27.5%; *SD* = 18.9) and shrub cover followed a similar pattern (range: 9.1–53.1%; *M* = 26%; *SD* = 8.3). The number of habitat patches per SU varied between 14 and 163 (*M* = 52.9; *SD* = 25.7), and patch shape was diverse (index range: from 1.3 [highly irregular forms] to 7.8 [regular forms]; *M* = 3.13; *SD *= 1.3). Some SUs included a relatively high length of edge (*c*. 48,000 m), whereas others had as little as 4,755 m.

### 3.2 Human–predator relations data and illegal killing prevalence across the landscape

A total of 233 respondents completed the questionnaire, of which 20% were women and 80% men. The median age of respondents was 55 years (interquartile range: 46–67). Participants had lived in their properties for 25–50 years (median = 35), which varied from 1 to 1,200 ha in size (median = 29). Land subdivision within SUs also varied widely (range: 1–314 properties; *M* = 41.3; *SD* = 37.2). Respondents, on average, received a monthly income equivalent to US$558 (*SD* = 2.81) and had completed 10 years of formal schooling.

Encounters with güiñas were rare. Nearly half of the respondents (49%, *n* = 116) reported seeing a güiña during their lifetime. However, on average, the sighting occurred 17 years ago (*SD* = 15). This percentage dropped to 10% and 21% during the last 4 (within the timeframe of the camera‐trap survey) and 10 years (time period for the RRT question) respectively. Predation events were also uncommon. Only 16% of respondents (*n* = 37) attributed a livestock predation event in their lifetime to a güiña, with just 7% (*n* = 16) stating that this had occurred in the past decade. Of the güiña predation events over the past decade (*n* = 16), 81% were recorded in Andean SUs.

When presented with scenario style questions concerning hypothetical livestock predation by a güiña, 38% (*n* = 89) of respondents stated that they would kill the felid if two chickens were lost, rising to 60% (*n* = 140) if 25 chickens were attacked. Using RRT, we found that 10% of respondents admitted to having killed a güiña in the last 10 years (*SE* = 0.09; 95% CI 0.02–0.18). The likelihood of a respondent admitting to killing güiña increased significantly with encounter frequency (β = 0.85, *SE* = 0.50; LRT ∆*G* ^2^ = 4.18, *p *=* *.04); those reporting the highest level of encounter rate were 2.3 times more likely to have killed the species compared to those not encountering güiña (Table 2). Data from the scenario‐based question on predation were excluded from the model due to a high β and associated standard error.

**Table 2:** The relationship between illegal killing of güiña (*Leopardus guigna*) and potential predictors of the behaviour. Reported coefficients, *SE*s, odds ratios and their 95% confidence intervals were derived from a multivariate logistic regression which incorporates the known probabilities of the forced RRT responses. Significance was accepted at the .05 level

	Coefficient	*SE*	*p*	Odds ratio	Odds ratio	
					Lower CI	Upper CI
(Intercept)	−2.43	1.99	.25	0.09	0.00	4.36
Age	−0.41	0.43	.38	0.66	0.29	1.54
Income	0.00	0.55	.99	0.99	0.34	2.96
Land parcel dependency	0.02	0.83	.98	12.02	0.20	5.19
Number of chicken holdings	−0.18	0.71	.78	0.83	0.21	3.38
Knowledge of legal protection	0.48	0.77	.57	1.62	0.36	7.37
Frequency of encounter	0.85	0.50	.04	2.34	0.87	6.28

### 3.3 Detection/non‐detection data

A total of 23,373 camera‐trap days returned 713 sampling occasions with a güiña detection (season 1 = 96; season 2 = 185; season 3 = 240; season 4 = 192). The naïve occupancy (i.e. proportion of sites with detection) was similar across all four seasons (0.54; 0.52; 0.58; 0.59) and between the central valley and Andean SUs (both areas >0.5). There was no evidence of spatial autocorrelation among SUs during any survey season (season 1 Moran's *I *=* *−0.03 [α = 0.74]; season 2 *I *=* *0.05 [α = 0.31]; season 3 *I *=* *0.05 [α = 0.36]; season 4 *I *=* *0.07 [α = 0.17]).

### 3.4 Integrated socioecological multiseason occupancy modelling

Our preliminary evaluation indicated that a Markovian dependence model structure was an appropriate description of the data. This dependence implies that güiña presence at a given site in a particular season is dependent on whether that site was occupied in the previous season (Table 3). Model 1.1 was chosen as the base structure for the modelling procedure because: (1) it is supported by AIC and (2) its parameterisation using extinction and colonisation (i.e. not derived parameters) allowed the role of different potential predictors to be tested on these population processes. Also, letting extinction and colonisation be season specific accommodated for unequal time intervals between sampling seasons.

**Table 3:** Seasonal occupancy dynamics models following MacKenzie et al. (2006), applied to the guiña (*Leopardus güigna*), to define the base model structure for the subsequent model selection procedure to evaluate potential habitat configuration/quality and human–predator predictors. Fitted probability parameters are occupancy (ψ), colonisation (γ), extinction (ε) and detection (*p*). Models assess whether changes in occupancy do not occur (model 1.6), occur at random (models 1.5, 1.4) or follow a Markov Chain process (i.e. site occupancy status in a season is dependent on the previous season; models 1.0, 1.1, 1.2, 1.3). Initial occupancy (ψ_1_) refers to occupancy in the first of four seasons over which the güiña was surveyed. Model selection procedure is based on Akaike's Information Criterion (AIC). ∆AIC is the difference in AIC benchmarked against the best model, *w*_*i*_ is the model weight, *K* the number of parameters and −2 × loglike is the value of the log likelihood at its maximum. The selected model is highlighted in bold

Model	Seasonal dynamic models	∆AIC	*w*_*i*_	*K*	−2 × loglike
1.0	ψ(.), γ(.), {ε= γ (1 − ψ)/ψ}, *p*(season)	0.00	0.443	6	3,982.93
**1.1**	**ψ**_**1**_ **(.), ε(season), γ(season),** ***p*** **(season)**	**0.36**	**0.370**	**11**	**3,973.29**
1.2	ψ_1_(.), ε(.), γ(.), *p*(season)	1.88	0.173	7	3,982.81
1.3	ψ_1_(.), ε(.), γ(.), *p*(.)	6.83	0.015	4	3,993.76
1.4	ψ_1_(.), γ(.),{ε = 1 − γ}, *p*(season)	41.78	0.000	6	4,024.71
1.5	ψ_1_(.), γ(season),{ε = 1 − γ}, *p*(season)	42.78	0.000	8	4,021.71
1.6	ψ(.), {γ = ε = 0}, *p*(season)	104.11	0.000	6	4,087.04

**Figure 3:**
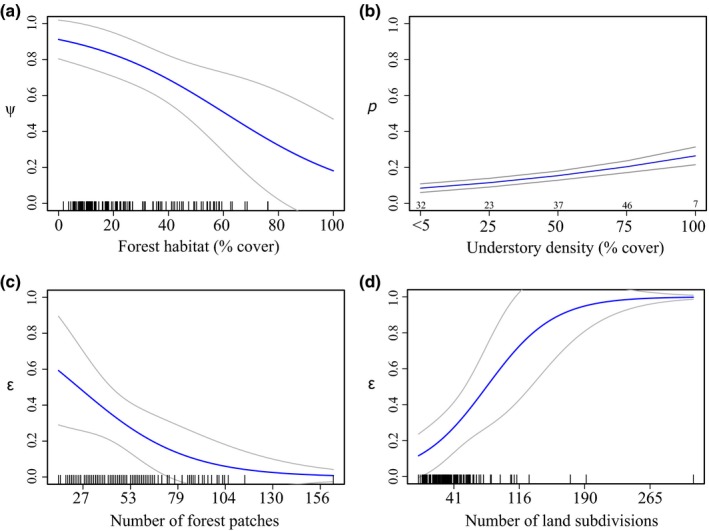
Predicted effects of forest cover, understorey density, number of habitat patches and land subdivision on multiseason occupancy model parameters for the güiña (*Leopardus guigna*). These results correspond to the final selected model [ψ_1_(Forest), *p*(season+Understorey), ε(season+PatchNo+Subdivision), γ(season)]. Grey lines delimit 95% confidence intervals. The tick marks along the *x*‐axis in (a), (c) and (d) indicate the underlying distribution of the continuous data. For (b), the small numbers above the *x*‐axis show the number of sites in each percentage cover class evaluated

Model selection for detection (models 2.1–2.7; Table 4) revealed a positive relationship with understorey vegetation cover (β_1_ = 0.343; *SE* = 0.055; Figure 3b). There was no evidence of an effect associated with the rotational camera‐trap survey design, and none of the other predictors were substantiated. Forest cover best explained initial occupancy (models 3.0–3.6; Table 4), with initial occupancy being higher in sites with less forest cover, although the estimated relationship was weak (β_1_ = −0.0363; *SE* = 0.0138; Figure 3a). Adding shrub cover only improved model fit marginally. Fragmentation metrics and land subdivision were not supported as good predictors.

**Table 4:** Multiseason models of initial occupancy (ψ_1_), extinction (ε), colonisation (γ) and detection (*p*) probability with potential habitat configuration/quality and human–predator predictors for the güiña (*Leopardus guigna*). Predictors were evaluated with a base model of seasonal dynamics [ψ_1_(.), ε(season), γ(season), *p*(season)] using a step‐forward model selection procedure and Akaike's information criterion (AIC). Initial occupancy (ψ_1_) refers to occupancy in the first of four seasons over which the güiña was surveyed, with occupancy dynamics following a Markov Chain process. ∆AIC is the difference in AIC benchmarked against the best model, *w*_*i*_ is the model weight, *K* the number of parameters and −2 × loglike is the value of the log likelihood at its maximum. The selected models for each parameter are highlighted in bold and used in the next step. ε was fitted first followed by γ, then vice versa

Model	Fitted parameter	∆AIC	*w*_*i*_	*K*	−2 × loglike
	*Detection/fitted with* ψ_*1*_ *(.),* ε*(season),* γ*(season)*				
**2.0**	***p*** **(season+Understorey)**	**0.00**	**0.9999**	**12**	**3,934.47**
2.1	*p*(season+Bamboo)	18.48	0.0001	12	3,952.95
	*Initial occupancy/fitted with* ε*(season),* γ*(season), p(season+Understorey)*				
**3.0**	**ψ**_**1**_ **(Forest)**	**0.00**	**0.5425**	**13**	**3,927.46**
3.1	ψ_1_(Forest+Shrub)	1.24	0.2918	14	3,926.7
3.4	ψ_1_(PatchNo)	4.00	0.0734	13	3,931.46
3.5	ψ_1_(.)	5.01	0.0443	12	3,934.47
3.6	ψ_1_(Subdivision)	5.69	0.0315	13	3,933.15
3.7	ψ_1_(Dogs)	7.00	0.0164	13	3,934.46
	*Extinction first/fitted with* ψ_*1*_ *(Forest), p(season+Understorey)*				
**4.0**	**ε(season+PatchNo), γ(season)**	**0.00**	**0.4692**	**14**	**3,920.10**
**4.1**	**ε(season+Subdivision), γ(season)**	**0.36**	**0.3919**	**14**	**3,920.46**
**4.2**	ε(season+PatchShape), γ(season)	5.15	0.0357	14	3,925.25
4.3	ε(season+Predation), γ(season)	5.24	0.0342	14	3,925.34
4.4	ε(season), γ(season)	5.36	0.0322	13	3,927.46
4.5	ε(season+FQencounter), γ(season)	5.92	0.0243	14	3,926.02
4.6	ε(season+FQPredation), γ(season)	7.24	0.0126	14	3,927.34
	*Colonisation second/fitted with* ψ_*1*_ *(Forest), p(season+Understorey) and 4.0/4.1 for* ε				
**4.7**	**ε(season+PatchNo), γ(season)**	**0.00**	**0.1877**	**14**	**3,920.10**
**4.8**	**ε(season+Subdivision), γ(season)**	**0.36**	**0.1568**	**14**	**3,920.46**
4.9	ε(season+Subdivision), γ(season+PatchShape)	0.79	0.1265	15	3,918.89
4.10	ε(season+PatchNo), γ(season+PatchShape)	1.29	0.0985	15	3,919.39
4.11	ε(season+Subdivision), γ(season+PatchNo)	1.63	0.0831	15	3,919.73
4.12	ε(season+PatchNo), γ(season+Edge)	1.84	0.0748	15	3,919.94
4.13	ε(season+PatchNo), γ(season+Forest)	1.98	0.0698	15	3,920.08
4.14	ε(season+Subdivision), γ(season+Edge)	2.16	0.0638	15	3,920.26
4.15	ε(season+ Subdivision), γ(season+Forest)	2.20	0.0625	15	3,920.30
4.16	ε(season+Subdivision), γ(season+Forest+Shrub)	3.50	0.0326	16	3,919.60
4.17	ε(season+PatchNo), γ(season+Forest+Shrub)	3.60	0.0310	16	3,919.70
4.18	ε(season), γ(season)	5.36	0.0129	13	3,927.46
	*Colonisation first/fitted with* ψ_*1*_ *(Forest), p(season+Understorey)*				
**5.0**	**ε(season), γ(season)**	**0.00**	**0.3303**	**13**	**3,927.46**
5.1	ε(season), γ(season+PatchShape)	0.96	0.2044	14	3,926.42
5.2	ε(season), γ(season+PatchNo)	1.55	0.1522	14	3,927.01
5.3	ε(season), γ(season+Edge)	1.89	0.1284	14	3,927.35
5.4	ε(season), γ(season+Forest)	1.95	0.1246	14	3,927.41
5.5	ε(season), γ(season+Forest+Shrub)	3.41	0.06	15	3,926.87
	*Extinction second/fitted with* ψ_*1*_ *(Forest), p(season+Understorey)* γ*(season)*				
**5.6**	**ε(season+PatchNo+Subdivision), γ(season)**	**0.00**	**0.8275**	**15**	**3,913.45**
5.7	ε(season+PatchNo), γ(season)	4.65	0.0809	14	3,920.10
5.8	ε(season+Subdivision), γ(season)	5.01	0.0676	14	3,920.46
5.9	ε(season+PatchShape), γ(season)	9.80	0.0062	14	3,925.25
5.10	ε(season+Predation), γ(season)	9.89	0.0059	14	3,925.34
5.11	ε(season), γ(season)	10.01	0.0055	13	3,927.46
5.12	ε(season+FQEncounters), γ(season)	10.57	0.0042	14	3,926.02
5.13	ε(season+FQPredation), γ(season)	11.89	0.0022	14	3,927.34

Model selection for extinction and colonisation (models 4.0–4.18 and 5.0–5.12; Table 4) reflected the same trends, irrespective of the order in which parameters were considered. Extinction, rather than colonisation, yielded predictors that improved model fit compared to the null model. Where predictors were fitted first on colonisation (models 5.0–5.5), none of the models tested improved fit substantially compared to the null model. This indicated that, of the available predictors, colonisation was only explained by seasonal differences. The human–predator predictors were not supported as drivers of either initial occupancy or extinction probability except for land subdivision (Table 4).

We fitted a final model (model 5.6; Table 4) with number of patches and land subdivision, which were identified as important predictors in the two top competing extinction models (models 5.7 and 5.8). This model was well supported. A goodness‐of‐fit test suggested lack of fit based on the global metric (*p*‐global<.05), but inspection of survey‐specific results show no such evidence (*p *>* *.05) apart from season 2 (*p *=* *.032). Inspecting the season 2 data, we found that the relatively large statistic value appeared to be driven by just a few sites with unlikely capture histories (i.e. <12 detections). Given this, and the fact that data from the other seasons do not show lack of fit, we deem that the final model explains the data appropriately. The model predicts that SU extinction probability becomes high (>0.6) when there are less than 27 habitat patches and more than 116 land subdivisions (β_1_ = −0.900; *SE* = 0.451 and β_1_ = 0.944; *SE* = 0.373, respectively; Figure 3c,d). Occupancy estimates were high across seasons with derived seasonal estimates of 0.78 (*SE* = 0.09), 0.64 (*SE* = 0.06), 0.80 (*SE* = 0.06) and 0.83 (*SE* = 0.06).

## 4 DISCUSSION

The integrated socioecological modelling framework we present here provides important insights into how habitat configuration/quality and human–predator relations may interact in space and time to effect carnivore populations existing across a human‐dominated landscape. We were able to disentangle the relative impact of a range of threats that have been highlighted previously in the literature as potential drivers of decline for our case study species the güiña.

The güiña is an elusive forest specialist. As such, one might predict that the species would be highly susceptible to both habitat loss and fragmentation (Ewers & Didham, 2006; Henle, Davies, et al., 2004). While the relationship between occupancy and higher levels of forest cover (Figure 3a) suggests that güiñas are likely to occupy areas with a large spatial extent of available habitat, our results also indicate that the species can tolerate extensive habitat loss. The effects of habitat loss could be confounded by time, and it is possible that we are not yet observing the impacts of this ecological process (Ewers & Didham, 2006). However, this is unlikely to be the case in this landscape as over 67% of the original forest cover was lost by 1970 and, since then, deforestation rates have been low (Miranda et al., 2015). Indeed, the findings highlight that intensive agricultural landscapes are very relevant for güiña conservation and should not be dismissed as unsuitable.

Spatially, the occupancy dynamics of this carnivore appear to be affected by fragmentation and human pressure through land subdivision. Ensuring that remnant habitat patches are retained in the landscape, and land subdivision is reduced so that existing bigger farms are preserved, could ultimately safeguard the long‐term survival of this threatened species. This should be the focus of conservation efforts, rather than just increasing the extent of habitat. Our findings further suggest that these remnant patches may play a key role in supporting the güiña in areas where there has been substantial habitat loss and, perhaps, might even offset local extinctions associated with habitat cover (Fahrig, 2002). A land sharing scheme within agricultural areas of the landscape could prove to be a highly effective conservation strategy (Phalan, Onial, Balmford, & Green, 2011) considering that these farms are currently not setting aside land, but are of high value to the species. The results also highlight that farmers with large properties are key stakeholders in the conservation of this species and must be at the centre of any conservation interventions that aim to protect existing native forest vegetation within farmland.

Following farming trends globally, larger properties in the agricultural areas of southern Chile are generally associated with high‐intensity production, whereas smaller farms are mainly subsistence‐based systems (Carmona, Nahuelhual, Echeverría, & Báez, 2010). It is therefore interesting, but perhaps counterintuitive, that we found occupancy to be higher (lower local extinction) where there is less land subdivision. However, a greater number of small farms are associated with higher human density which may result in increased persecution by humans (Woodroffe, 2000). Also, higher subdivision imposes pressure on natural resources, due to more households being present in the landscape (e.g. Liu, Daily, Ehrlich, & Luck, 2003), which has been shown to reduce the quality of remaining habitat patches as a result of frequent timber extraction, livestock grazing (Carmona et al., 2010) and competition/interference by domestic animals and pets (Sepúlveda, Singer, Silva‐Rodríguez, Stowhas, & Pelican, 2014). Native vegetation in non‐productive areas, including ravines or undrainable soils with a high water‐table, is normally spared within agricultural areas (Miranda et al., 2015), and these patches of remnant forest could provide adequate refuge, food resources and suitable conditions for carnivore reproduction (e.g. Schadt et al., 2002). However, it is possible that areas with high land subdivision and a large number of patches could be acting as ecological traps if source–sink dynamics are operating in the landscape (Robertson & Hutto, 2006). Additionally, another factor driving the subdivision of land and degradation of remnant forest patches across agricultural areas is the growing demand for residential properties (Petitpas, Ibarra, Miranda, & Bonacic, 2017). This is facilitated by Chilean law, which permits agricultural land to be subdivided to a minimum plot size of 0.5 ha. Furthermore, it is a common practice for sellers and buyers to completely eliminate all understorey vegetation from such plots (C. Rios, personal communication) which, as demonstrated by detection being higher in dense understorey, is a key component of habitat quality. The fact that farmers subdivide their land for economic profit, driven by demand for residential properties, is a very complex and difficult issue for future landscape‐level conservation.

Although previous studies have suggested that human persecution may be a factor contributing to the decline of the güiña (Nowell & Jackson, 1996; Sanderson et al., 2002), illegal killing in the study region appears low and much less of a threat to the species than the habitat configuration in the landscape. Although the species occupies a large proportion of the landscape across seasons, people report that they rarely encounter the carnivore or suffer poultry predation. The güiña's elusive behaviour is reinforced by our low camera‐trap detection probability (*p *<* *.2 over 2 nights). One in 10 respondents (10%) admitted to killing a güiña over the last decade. One potential drawback of RRT is that it is impossible to know if people are following the instructions (Lensvelt‐Mulders & Boeije, 2007). However, we deployed a symmetrical RRT design (both “yes” and “no” were assigned as prescribed answers), which increases the extent to which people follow the instructions (Ostapczuk & Musch, 2011). Moreover, the proportion of “yes” answers in the data exceeded the probability of being forced to say “yes” (which in this study was 0.167), indicating that respondents were reporting illegal behaviour. From our data, it would be difficult to determine whether this prevalence of illegal killing has a detrimental impact on the population size of the species. However, with our framework, we could, in the future, evaluate spatial layers of information such as the probability of illegal killing based on the distribution of encounters with the güiña and landscape attributes that increase extinction probability (e.g. land subdivision and reduced habitat patches) in order to be spatially explicit about where to focus conservation and research efforts (e.g. Santangeli et al., 2016).

Our results demonstrate the benefits of integrating socioecological data into a single modelling framework to gain a more systematic understanding of the drivers of carnivore decline. The framework teased apart the relative importance of different threats, providing a valuable evidence base for making informed conservation recommendations and prioritising where future interventions should be targeted for the case study species. Prior to applying our framework, conservationists believed that human persecution was instrumental in determining güiña occupancy patterns in human‐dominated landscapes. However, our combined socioecological approach highlighted that habitat configuration/quality characteristics are the primary determinants, mainly due to the widespread presence of the species across the landscape and lack of interaction with rural homes. The relative importance of, and balance between, social and ecological factors may differ according to the species of conservation concern. While our framework might not be to resolve conflict, it can help guide potential stakeholder controversies (Redpath et al., 2013, 2017) by improving our understanding of how carnivores interact with humans in space and time (Pooley et al., 2016). A number of small to medium carnivores in need of research and conservation guidance (Brooke, Bielby, Nambiar, & Carbone, 2014) could benefit from our framework.

## AUTHORS’ CONTRIBUTIONS

All authors conceived ideas and designed methodology. N.G. collected and processed data. N.G. and Z.G.D. led the writing of the manuscript. All authors contributed critically to drafts and have given their approval for publication.

## DATA ACCESSIBILITY

Data available from the Dryad Digital Repository https://doi.org/10.5061/dryad.qt0jq (Gálvez et al., 2018).
