# Supplementary material for: A spatially integrated framework for assessing socioecological drivers of carnivore decline
Source: J Appl Ecol. 2018 Jan 15;55(3):1393–405. doi: 10.1111/1365-2664.13072 (PMC5947557; doi:10.1111/1365-2664.13072)
Supplement: Supplementary file 1 [file JPE-55-1393-s001.docx]

**Supporting Information**

**Appendix S1:** **Landcover classification of study area**

Landcover classification was carried out using a composite of four Aster images at 15 m resolution from between 2002 and 2007. Native forest cover within the study region did not change significantly between 1983 and 2007 (Petitpas 2017; Miranda *et al.* 2015). In addition, the current extent and configuration of forest across the sample units (SUs) has not altered perceptibly when compared visually with up-to-date Google Earth imagery from 2014. The study region was categorised into nine landcover classes ((i) water; (ii) forest, (iii) forest regrowth, (iv) shrub/bog, (v) grassland, (vi) hualve (inundated forests), (vii) plantation, (viii) crop/pasture/orchard and (ix) bare ground/sand/lava rock) using a supervised classification with maximum likelihood estimation, based on field data from 738 training points. A further 738 points were used to verify classification accuracy, which was ‘almost perfect’ (Kappa= 0.81 (SE= 0.017); Landis & Koch 1977; Congalton 1991). Urban landcover digitised by hand and added as a tenth class. Image processing and classification were conducted in ERDAS Imagine 2014 (Hexagon Geospatial, Norcross, GA, USA) and ArcMap v.10.1 (ESRI, Redlands, CA, USA).

**Appendix S2:** **Generation of the human-predator relations data, used as potential predictors to model multi-season occupancy dynamics of the guiña** **(*Leopardus guigna*)**

The questionnaire delivery and design were approved by School of Anthropology and Conservation Research and Research Ethics Committee, University of Kent, as well as the Villarrica Campus Committee of the Pontificia Universidad Católica de Chile. All householders were fully informed of the study objectives, but with care taken to ensure that the information provided would not lead to (un)conscious bias in the participant’s responses. The contact and employment details for the principal researcher were provided in case any unforeseen issues were experienced after completing the questionnaire. The respondents were told that their engagement in the research was entirely voluntary and that they could withdraw from the process at any point, without needing to provide an explanation. Additionally, they were notified that their answers to the questionnaire would be anonymised and only ever presented in aggregate form, so their identity would not be discernible. The respondents were also assured that the data would be stored securely, only accessible by the lead researcher and would not be passed on to any second parties, in line with the UK Data Protection Act. Each individual was then given time to evaluate all this information, prior to signing an informed consent sheet.

The questionnaire consisted of six sections. The first part included socio-demographic/economic questions relating to age, amount of schooling, livelihood activities and income. The next section focussed on questions regarding killing wild animals, including species with protected (e.g. puma, guiña) and non-protected status (e.g. introduced wild boar). To prevent any bias in responses, our questions included all native carnivores known to occur across the study region, as well as free-roaming domestic dogs. As killing of protected species is an illegal activity, we employed the Randomised Response Technique (RRT) described in St John et al. (2010). A dice was used as randomisation tool; respondents were asked to provide a truthful answer if they rolled a one, two, three or four, must answer “yes” if they rolled a five (irrespective if it is true answer or not) and must answer “no” if the dice landed on six. The time period used to provide context to the question was ‘over the last ten years’, which was deemed most appropriate after the pilot exercise. Trial runs were conducted using non-sensitive questions to ensure the RRT instructions were understood and being followed by the respondents. A visual barrier was used to ensure that the interviewer could not see the number on the rolled dice.

The third part of the questionnaire asked respondents to report livestock losses via predation over the past year, or an alternative time period they could quantify. In the fourth section, participants were probed about their knowledge of whether the hunting of each species was permitted or illegal, as well as asking how frequently the species were encountered. A fifth section aimed to evaluate scenarios of predation with a hypothetical livestock holding of 100 sheep and chickens. Respondents were asked what behaviour they would display towards the carnivores occurring in the study region after a specific level of predation (2, 10, 25, 50, >50 sheep or chickens) has been experience. For sheep predation, we assessed the puma (*Puma concolor*) and domestic dogs (*Canis familiaris*), and for chicken predation we asked about guiña and Harris hawk (*Parabuteo unicinctus*). In order not to bias responses, respondents were offered a choice of possible actions (e.g. lethal controls, call authorities, improve management, nothing, etc.). The value of this hypothetical predation scenario was interpreted as a measure to tolerance to predation. The final section centred on the management of livestock, particularly sheep and chickens, in relation to behaviour such as enclosing livestock at night, the distance of the closure from household, the number of domestic dogs/cats associated with the property and how they are managed overnight (e.g. free-roaming, tethered), as well as how often they are fed and the type of food they are given.

The original (Spanish) and translated (English) questions were as follows:

| **RANDOMISED RESPONSE (RRT)** | **Response Type** |
| --- | --- |
| 1. During the last 10 years, have you killed a wildboar?   En los últimos diez años ha matado a un Jabalí? | Yes/No |
| 1. During the last 10 years, have you killed a puma?   En los últimos diez años ha matado a un puma? | Yes/No |
| 1. During the last 10 years, have hired someone to kill a puma?   En los últimos diez años ha matado a contratado a alguien para matar a un puma? | Yes/No |
| 1. During the last 10 years, have you killed a guiña?   En los últimos diez años ha matado a una guiña? | Yes/No |
| 1. During the last 10 years, have you killed a fox?   En los últimos diez años ha matado a un zorro? | Yes/No |
| 1. During the last 10 years, have you killed a hawk?   En los últimos diez años ha matado a un peuco? | Yes/No |
| 1. During the last 10 years, have you killed a rabbit or hare?   En los últimos diez años ha matado a un conejo o liebre? | Yes/No |
| 1. During the last 10 years, have you killed a free roaming domestic dog not of your ownership?   En los últimos diez años ha matado a un perro doméstico andariego que no es de su propiedad? | Yes/No |
| 1. During the last 10 years, have you killed a weasel?   En los últimos diez años ha matado a un quique? | Yes/No |
| 1. During the last 10 years, have you killed a skunk?   En los últimos diez años ha matado a un chingue? | Yes/No |
| **HOUSEHOLD INFORMATION** | |
| 1. What is the size of your property in hectares?   Cuál es el tamaño de su propiedad? | *Exact figure* |
| 1. How long have you lived here? Where are you originally from?   Hace cuánto vive en el sector? De donde es? | *Exact figure* |
| 1. What is your age? And that of other adults in the household?   Cuál es la edad de los adultos del hogar? (dueños de casa) | *Exact figure* |
| 1. What is your level of schooling? And that of other adults in the household?   Cuál es el nivel escolar de los adultos del hogar? (dueños de casa) | *Exact figure* |
| 1. How many children do you have?   Cuantos hijos tiene? | *Exact figure* |
| 1. Please classify in order of importance the following economic activities for your overall income?   Clasifique en orden de importancia para su ingreso familiar las siguientes actividades económicas? | Crops/Livestock/Forestry/Urban services/ Agricultural services/Tourism/Subdivision of land for residential development/Other |
| 1. What is your approximate monthly income?   Cuál es su ingreso mensual aproximado? | *Exact figure* |
| **PREDATION OF DOMESTIC ANIMALS** | |
| 1. What are your livestock animal holdings during the past year?   Cuantos animales ha tenido durante el año pasado? | Bovine/Ovine/Chickens/Others |
| 1. How many livestock animals have you lost because of this predator in the past year? If respondent could not quantify over the past year their alternative time period was noted (e.g. 3 sheep killed by puma in 5 years)   Cuántos animales ha perdido por parte del predador? Si el entrevistado no podía cuantificar en un año, entonces se anotaba el periodo de tiempo en el cual sufrió un numero de pérdida (e.g. 3 ovejas predadas por puma en 5 años)  *The question was repeated in turn for the following predators: puma, guiña, fox, hawk, domestic dogs, skunk, weasel*  *La pregunta fue repetida para puma, guiña, zorros, peucos (rapaces diurnas), perros domésticos, chingues y quique.* | *Exact figure* |
| **KNOWLEDGE OF PREDATOR LEGAL STATUS** | |
| 1. From your knowledge, is hunting this predator prohibited?   Según su conocimiento, se puede cazar al animal?  *The question was repeated in turn for the following predators:* puma, guiña, fox, hawk, domestic dogs, skunk, weasel, hare-rabbit  La pregunta fue repedita para puma, guiña, zorros, peucos (rapaces diurnas), perros domésticos, chingues, quique y liebre y conejos | Yes/No/Do not know |
| **FREQUENCY OF PREDATOR ENCOUNTERS** | |
| 1. How frequently do you observe a sign or sound indicating that this predator has been on your property? Please use a unit of time that you can remember (daily, weekly, monthly, yearly)   Con que frecuencia observa (o algún indicio) al animal en su propiedad? Use una medida de tiempo que recuerde (diario, semanal, mensual, anual).  *The question was repeated in turn for the following predators:* puma, guiña, fox, hawk, domestic dogs, skunk, weasel, hare-rabbit  La pregunta fue repedita para puma, guiña, zorros, peucos (rapaces diurnas), perros domésticos, chingues, quique y liebre y conejos | *Exact figure* |
| **SCENARIO-BASED QUESTION: HYPOTHETICAL RESPONSE TO PREDATION** | Open ended question with internal codes for:  (1)Call authorities; (2)Intent to hunt it; (3)Capture and call authorities; (4)Scare off; (5)Nothing; (6)Observe; (7)Protect my livestock holdings; (8)other |
| “Let’s suppose that you have 100 sheep” / “Digamos que usted tiene 100 ovejas” | |
| 1. What do you think you would do if the puma kills X/100 Sheep   Qué haría si un puma le mata X/100 ovejas?  X = 2, 10, 25, 50, >50 | Internal code |
| 1. What do you think you would do if a domestic dog kills X/100 sheep   Qué haría si un perro doméstico le mata X/100 ovejas?  X = 2, 10, 25, 50, >50 | Internal code |
| “Let’s suppose that you have 100 Sheep” / “Digamos que usted tiene 100 Ovejas” | |
| 1. What do you think you would do if the guiña kills X/100 chickens?   Qué haría si un guiña le mata X/100 chickens?  X = 2, 10, 25, 50, >50 | Internal code |
| What do you think you would do if a hawk kills X/100 chickens?  Qué haría si un peuco (todas las rapaces diurnas) le mata X/100 gallinas?  X = 2, 10, 25, 50, >50 | Internal code |
| **DOMESTIC ANIMAL MANAGEMENT** | |
| 1. How do you keep your livestock animals at night?   Como guarda sus animales durante la noche?  Question asked for sheep and chickens  Pregunta realizada para ovejas y gallinas | Closed housing/Open corral/Open field with dog/Open field without dog/Other, how? |
| 1. At what distance do you keep your livestock animals at night? meters   A que distancia de su casa guarda sus animales durante la noche? metros  Question asked for sheep and chickens  Pregunta realizada para ovejas y gallinas | *Exact figure* |
| 1. How many dogs/cats do you have?   Cuantos perros/gatos tienen en su casa? | *Exact figure* |
| 1. What do you do with your dogs/cats at night?   Que hace con sus perros/gatos durante la noche? | Enclosure/Tied/Free-roaming/Other |
| 1. With what do you feed your dog/cat?   Con que alimenta a sus perros/gatos? | Commercial pellets/Kitchen scraps/Mix of pellets and kitchen scraps/Grain/Mix of grain and kitchen scraps/Nothing/Other |

**Table S1:** Description of potential habitat configuration/quality and human-predator predictors used when modelling initial occupancy (ψ_1_), colonisation (γ), extinction (ε) and detection (*p*) probability parameters from multi-season camera-trap surveys of the guiña (*Leopardus guigna*). Detailed description of habitat configuration metrics can be found in (McGarigal *et al.* 2002).

| **Predictor** | **Abbreviation**  **in models** | **Description**^§§^ |
| --- | --- | --- |
| *Habitat configuration* |  |  |
| Percent forest cover | Forest | Metric that measures habitat loss as the extent of forest cover in a sample unit (0-100). Forest cover was obtained by pooling old-growth and secondary forest landcover classes, which are both considered to be suitable guiña habitat (Nowell & Jackson 1996; Acosta-Jamett & Simonetti 2004). |
| Percent shrub cover | Shrub | Metric that measures the extent of shrub cover in a sample unit (0-100). The spatial configuration is not assessed because shrub is a marginal habitat and evaluated for an additive effect on forest cover. As shrub can be considered a marginal habitat for guiña (Dunstone *et al.* 2002; Sanderson, Sunquist & W. Iriarte 2002; Acosta-Jamett & Simonetti 2004), we also measured the extent of shrub cover to evaluate possible additive effects with habitat cover |
| Number of forest patches | PatchNo | Metric that measures the number of forest habitat patches (0-∞). |
| Shape index forest patches | PatchShape | Shape metric that measures the complexity of forest habitat patch shape compared to a square, weighted for the entire landscape. As the index value increases, that habitat patch shape is more irregular (1-∞). |
| Forest patch size area^†^ | PatchAreaW | Metric that measures mean habitat patch area (0-∞) corrected for sample unit scale. It provides a landscape centric perspective of patch structure. |
| Forest patch continuity^†^ | Gyration | Metric that measures habitat patch continuity (0-∞). It can be interpreted as the average distance an organism can move within the habitat before an edge is encountered (McGarigal *et al.* 2002). The value increases with greater habitat patch extent. |
| Edge length of forest | Edge | Area-edge metric that measures the total length (0-∞) of habitat patch edge across a sample unit. This can be used instead of edge density because we are comparing sample units of the same size (McGarigal *et al.* 2002). The value rises with increasing edge. |
| Landscape shape index of forest^‡^ | LSI | Aggregation metric that compares the landscape level edge of the habitat to one without internal edges or a square (0-100). This is a measure of the level of fragmentation in a sample unit. |
| Patch Cohesion^†^ | COH | Aggregation metric that measures the physical connectedness (0-1) of forest habitat cover by measuring the aggregation of patches. |
| *Human-predator relations data* | |  |
| Land subdivision | Subdivision | Measures the number of land tenure divisions (i.e. owners) in a sample unit (0-∞). We expect higher subdivision to represent greater anthropogenic pressure and management variability from factors such as logging and presence of domestic dogs which were not measured directly in each sample unit (e.g. Theobald, Miller & Hobbs 1997; Hansen *et al.* 2005; Western, Groom & Worden 2009). Subdivision was based on the number of properties or land parcels recorded in each SU from national records (CIREN-CORFO, 1999). |
| Intent to kill | Intent | Intent to kill guiña by households in a sample unit (categorical: yes= 1, no= 0). This measure describes how a respondent states they would respond if a guiña two of their chickens. It is a highly conservative indicative measure of tolerance to livestock predation before lethal control is considered. |
| Predation | Predation | Occurrence of chicken predation by guiña in a sample unit (categorical: yes= 1, no= 0). |
| Frequency of predation | FQPredation | Frequency of chicken predation by guiña in a sample unit. Predation events were scaled to yearly frequency (0-∞). |
| Frequency of encounter^§^ | FQEncounter | Numbers of encounters householders have had with guiña, scaled to a yearly frequency (0-∞). Frequency of encounters is also used to fit detection probability as a proxy for the elusiveness of the species. |
| Number of dogs | Dogs | Maximum number of free-roaming dogs, owned by the household, at night in proximity to the camera-traps (0-∞). We assume this value to be a conservative proxy to dog activity and an index of interference/competition by dogs. We also fitted extinction probability with free roaming dogs as they have been documented to interfere and kill wildlife in Chile (Silva-Rodriguez, Ortega-Solis & Jimenez 2010; Silva-Rodríguez & Sieving 2012), therefore we included average number of free roaming domestic dogs of nearby households (from our questionnaire Appendix S2 as a potential source of mortality. Because guiña are mainly nocturnal (Delibes-Mateos *et al.* 2014; Hernandez *et al*. 2015) we excluded households that restrain dogs at night. |
| *Habitat quality and survey specific variables*^§^ |  |  |
| Bamboo density  (*Chusquea* spp.) | Bamboo | Bamboo density (*Chusquea* spp.) within a 25 m radius of each camera-trap, recorded in five categorical percentage classes (Braun-Blanquet 1965). |
| Density of understory | Understory | Understory vegetation density within a 25 m radius of each camera-trap, recorded in five categorical percentage classes (Braun-Blanquet 1965). |
| SU rotation | Rotation | Each SU was included in one of four consecutively sampled rotations of camera-traps during each season. |
| Intensity of livestock activity | Livestock | Livestock activity next to each camera-trap visually assessed and recorded using three categories (high, medium or low intensity). Based on signs such as presence of animals, grazed vegetation, trampled paths and manure. |
| Intensity of logging activity | Logging | Logging activity next to each camera-trap visually assessed and recorded using three categories (high, medium or low intensity). Based on signs such as active firewood piles, clearings, logging paths, fresh stumps and fallen logs. |
| Water availability | Water | The availability of water was recorded as either present or absent at the patch level during each season (categorical: yes= 1, no= 0). |

^†^Predictor excluded due to collinearity with percent of forest cover (Pearson’s│r│>0.7)

^‡^Predictor excluded due to collinearity with number of forest patches (Pearson’s│r│>0.7)

^§^Predictors fitted only with detection probability at the forest patch level

^§§^ Supporting information references:

Braun-Blanquet, J. (1965) Plant Sociology: The Study of Plant Communities. Hafner, London.

CIREN (Centro de Información de Recursos Naturales), CORFO (Corporación de Fomento), 1999. Digital Cartography of Rural Properties.

Congalton, R.G. (1991) A review of assessing the accuracy of classifications of remotely sensed data. Remote Sensing of Environment, 37, 35–46.

Delibes-Mateos, M., Díaz-Ruiz, F., Caro, J. & Ferreras, P. (2014) Activity patterns of the vulnerable guiña (*Leopardus guigna*) and its main prey in the Valdivian rainforest of southern Chile. Mammalian Biology, 79, 393–397.

Hansen, A.J., Knight, R.L., Marzluff, J.M., Powell, S., Brown, K., Gude, P.H. & Jones, K. (2005) Effects of exurban development on biodiversity: patterns, mechanisms, and research needs. Ecological Applications, 15, 1893–1905.

Hernandez, F., Galvez, N., Gimona, A., Laker, J. & Bonacic, C. (2015) Activity patterns by two colour morphs of the vulnerable guiña *Leopardus guigna* (Molina 1782), in temperate forests of southern Chile. Gayana, 79, 102–105.

Landis, J.R. & Koch, G.G. (1977) The measurement of observer agreement for categorical data. Biometrics, 33, 159–174.

Silva-Rodriguez, E., Ortega-Solis, G.R. & Jimenez, J.E. (2010) Conservation and ecological implications of the use of space by chilla foxes and free‐ranging dogs in a human‐dominated landscape in southern Chile. Austral Ecology, 35, 765–777.

Silva-Rodríguez, E.A. & Sieving, K.E. (2012) Domestic dogs shape the landscape-scale distribution of a threatened forest ungulate. Biological Conservation, 150, 103–110.

St John, F.A. V, Edwards-Jones, G., Gibbons, J.M. & Jones, J.P.G. (2010) Testing novel methods for assessing rule breaking in conservation. Biological Conservation, 143, 1025.

Theobald, D.M., Miller, J.R. & Hobbs, N.T. (1997) Estimating the cumulative effects of development on wildlife habitat. Landscape and Urban Planning, 39, 25–36.

Western, D., Groom, R. & Worden, J. (2009) The impact of subdivision and sedentarization of pastoral lands on wildlife in an African savanna ecosystem. Biological Conservation, 142,

2538–2546.
